# Methodological Appraisal of the Munro Scale: Psychometric Properties and Diagnostic Accuracy in Adult Surgical Patients: A Methodological Study

**DOI:** 10.1002/hsr2.72464

**Published:** 2026-04-28

**Authors:** Leila Sadati, Sahar Karami, Nasim Jamshid Malekara, Rana Abjar, Cassendra A. Munro, Niloofar Hajati

**Affiliations:** ^1^ Department of Operating Room, School of Paramedical Sciences Alborz University of Medical Sciences Karaj Iran; ^2^ Department of Operating Room, School of Paramedical Sciences Tehran University of Medical Sciences Tehran Iran; ^3^ Department of Anesthesia & Surgical Technology, School of Allied Medical Sciences Urmia University of Medical Sciences Urmia Iran; ^4^ Stanford Health Care Stanford University Stanford California USA; ^5^ Department of Operating Room, TMS.c. Islamic Azad University Tehran Iran

**Keywords:** Munro scale, perioperative risk factors, pressure injury, validity‐reliability

## Abstract

**Background:**

Pressure injuries during the perioperative period can adversely affect postoperative outcomes and patient recovery. An essential component of prevention and patient care is systematic risk assessment. The Munro Pressure Ulcer Risk Assessment Scale is a perioperative‐specific tool designed to evaluate patients' risk for pressure injuries, enabling early identification of high‐risk individuals and facilitating timely preventive interventions by perioperative nurses.

**Aim:**

This methodological study aimed to evaluate the validity, reliability, and diagnostic Accuracy of the Munro Pressure Ulcer Risk Assessment Scale (Munro Scale) in adult patients undergoing surgery.

**Method:**

This methodological study was conducted on a sample of 200 adult surgical patients from October 2024 to May 2025. Data were collected using the Munro Pressure Injury Risk Assessment Scale alongside a demographic questionnaire. The scale underwent a rigorous process of translation and cultural adaptation following established methodological standards. Psychometric testing encompasses evaluations of content validity, construct validity, and Cronbach's alpha coefficient.

**Result:**

Out of 200 surgical patients, 37 (18.5%) developed pressure injuries, mostly classified as Stage I and Stage II. Confirmatory factor analysis (CFA) was conducted using AMOS to evaluate the construct validity of the scale, which included three latent factors: preoperative, intraoperative, and postoperative risk factors. Factor loadings for most items exceeded 0.4, indicating acceptable item relevance. Reliability analysis showed Cronbach's alpha values of 0.84, 0.62, and 0.83 for the preoperative, intraoperative, and postoperative factors, respectively. Composite reliability (CR) values were 0.83, 0.71, and 0.82, confirming acceptable internal consistency.

**Conclusion:**

The Munro Scale demonstrated acceptable reliability and validity, confirmatory factor analysis indicating a moderate or limited fit. This scale can be used to assess the risk of pressure injuries in perioperative patients.

## 1 Introduction

Pressure injuries are localized injuries to the skin or underlying tissue over bony prominences, resulting from sustained pressure, shear, or friction [1]. Pressure injury is one of the common complications in hospitalized patients, especially in those undergoing surgery [2]. A systematic review reported surgery‐related pressure ulcer incidences ranging from 0.3% to 57.4% [3], and Iranian studies have found an average prevalence of 18.96% [4]. Sensory loss and immobility during and after surgery heighten patients' vulnerability, making the intraoperative phase especially critical for prevention [5].

Risk factors for pressure injuries can be grouped into three categories: intrinsic (patient‐related): malnutrition (albumin < 3 g/dL), advanced age, immobility, and comorbidities such as diabetes and peripheral vascular disease [6]. Extrinsic: ambient temperature, friction, shear forces, and moisture [7]. Surgery‐related: prolonged operative time, patient positioning, and type of anesthesia [8, 9].

Prolonged procedures and the inability to reposition patients impose continuous pressure on bony prominences, leading to significant tissue damage [10]. Preventive techniques such as micro‐movements and optimized pressure distribution have demonstrated efficacy in reducing intraoperative ulcer risk [11, 12, 13]. Early identification of at‐risk patients is essential, given the high costs and complications of treating pressure ulcers [14, 15]. A reliable assessment tool enables perioperative teams to implement targeted interventions and minimize pressure injury development [16].

The Munro scale, developed by a perioperative nurse in 2010, evaluates pressure injury risk across three phases: preoperative, intraoperative, and postoperative, with 15 items per phase scored from 1 to 3 [17]. It incorporates surgery‐specific factors (anesthesia type, positioning, hemodynamic fluctuations, blood loss) that are not captured by general scales like the Braden scale [16, 18].

Although the Munro scale has been translated and culturally adapted in Turkey, Italy, and Brazil [14, 19, 20], its psychometric properties have not been assessed in Persian. In this study, we evaluated the Munro scale in terms of its sensitivity, specificity, accuracy, positive predictive value (PPV), and negative predictive value (NPV). To our knowledge, no previous studies have assessed these diagnostic performance characteristics of the Munro scale in surgical patients.

## 2 Materials and Methods

### 2.1 Study Design

This methodological study aimed to evaluate the validity and reliability of the Munro Pressure Injury Risk Assessment Scale in adult patients undergoing surgery at three teaching hospitals affiliated with Alborz University of Medical Sciences with IR.ABZUMS.REC.1403.114 ethics code. Data collection occurred between October 2024 and May 2025. Informed consent was obtained from all participants prior to their enrollment in the study. The Munro scale comprises three distinct phases of assessment: preoperative, intraoperative, and postoperative.

Preoperative indicators: Six indicators were assessed prior to surgery: mobility, nutritional status, body mass index (BMI), weight loss, age, and comorbidities. The cumulative score ranges from 5 to 21, with risk levels interpreted as follows: A score of 5–6 indicates low or no risk, a score of 7–14 indicates moderate risk, and a score of 15 or higher indicates high risk.

Intraoperative indicators: Seven intraoperative factors were evaluated: ASA physical status classification, anesthesia type, body temperature, hypotension, moisture, surface/movement, and patient positioning. Combined with the preoperative score, the intraoperative score ranges from 12 to 42: A score of less than 13 indicates low risk, a score of 14–24 indicates moderate risk, and a score of 25 or higher indicates high risk.

Postoperative indicators: Two postoperative variables were considered: duration of stay in the operating room and volume of blood loss. The total score across all three phases ranges from 14 to 48: a score of less than 15 indicates low or no risk, a score of 16–28 indicates moderate risk, and a score of greater than 28 indicates high risk.

### 2.2 Sampling Method and Sample Size

Following Kline's (2016) recommendations for instrument validation, a sample of 200 patients was recruited using convenience sampling [5].

Patients were eligible for the study if they were 18 years of age or older, scheduled for elective surgery, and showed no signs of pressure ulcers, erythema, or skin redness at admission or prior to surgery. Additionally, only patients who provided informed consent and were willing to participate in the study were included. Patients were excluded from the study if they withdrew their consent at any point or if the assessment scale was not completed in its entirety.

### 2.3 Instrument Validation Process

The localization and validation of the Munro scale followed rigorous methodological standards, including translation, face/content/construct validity, and reliability testing.

#### 2.3.1 Translation and Back‐Translation

With formal permission from Dr. Munro, the original English version was translated into Persian. Two independent translators conducted back‐translations. The finalized Persian version was reviewed and approved by Dr. Munro.

#### 2.3.2 Face Validity

Quantitative assessment:

Fourteen surgical team members rated item importance on a 5‐point Likert scale (1 = “not important” to 5 = “extremely important”). Item Impact scores were calculated using the formula [21]:

Item impact=Frequency(%)×Mean importance

Qualitative assessment:

Twenty purposively selected surgical team members evaluated the clarity, cultural relevance, and placement of items.

#### 2.3.3 Content Validity

Qualitative assessment:

Ten experts in surgery, operating room practice, and psychometrics assessed item clarity and simplicity.

Quantitative assessment:

Content validity was evaluated using the Content Validity Ratio (CVR) and the Content Validity Index (CVI). CVR was calculated according to Lawshe's method, with all items scoring between 0.71 and 1.00, exceeding the recommended threshold of 0.62 for a panel of 10 experts. The CVI ranged from 0.81 to 1.00, with a mean CVR of 0.81 and a mean CVI of 0.89, indicating strong content validity for the scale [22].

#### 2.3.4 Construct Validity

Confirmatory Factor Analysis (CFA) was performed using AMOS software. The model included three latent constructs corresponding to the preoperative, intraoperative, and postoperative phases. Convergent validity was assessed via Average Variance Extracted (AVE), and model fit was evaluated using standard goodness‐of‐fit indices.

### 2.4 Reliability Analysis

Internal consistency and composite reliability were assessed using Cronbach's alpha and CR:

For the perioperative phases, the Cronbach's *α* and Composite Reliability (CR) values were as follows: preoperative phase, *α* = 0.84 and CR = 0.83; intraoperative phase, *α* = 0.62 and CR = 0.71; and postoperative phase, *α* = 0.83 and CR = 0.82. All values met acceptable thresholds [23].

### 2.5 Diagnostic Accuracy of the Munro Scale

The predictive accuracy of the scale was evaluated under two scenarios. In Scenario 1, only “high risk” scores were considered predictive of pressure ulcers, whereas in Scenario 2, both “High” and “moderate risk” scores were regarded as predictive. Diagnostic metrics calculated included accuracy (the proportion of correct predictions), sensitivity (true positive rate), specificity (true negative rate), positive predictive value (PPV), and negative predictive value (NPV).

### 2.6 Data Analysis

Data were analyzed using SPSS version 20 and AMOS version 23 software. Descriptive statistics (mean ± SD, frequency, and percentage) were used to summarize the data. Construct validity was evaluated using CFA, and model fit was assessed using *χ*²/df, RMSEA, RMR, CFI, GFI, and AGFI indices. Internal consistency was examined using Cronbach's alpha and CR, and convergent validity was assessed using AVE. Associations between categorical variables were analyzed using the *χ*² test. Diagnostic performance was evaluated by calculating sensitivity, specificity, accuracy, PPV, and NPV. A *p* value < 0.05 was considered statistically significant.

## 3 Result

### 3.1 Participant Demographics and Clinical Characteristics

Two hundred patients participated in the study. The mean age was 45.16 ± 17.64 years. Of these, 52.5% (*n* = 105) were male, and 47.5% (*n *= 95) were female. Surgical positioning and duration of surgery time and recovery time are summarized in Table 1. The distribution of pressure injury among participants, according to their anatomical location, is also presented in Table 2.

**Table 1:** The demographic and clinical characteristics of the enrolled patients (*n* = 200).

Variables	Number (%)/Mean ± SD (Range)	CI (95%)
Gender	200	
Male	105 (47.5%)	45.7–59.3
Female	95 (52.5%)	40.7–54.3
Age	45.16 ± 17.64 (17–88)	—
Surgery position		
Prone/supine	106 (53.0%)	46.0–60.0
Lateral	39 (19.5%)	14.0–25.0
Lithotomy	55 (27.5%)	21.0–34.0
Type of Surgery		
Orthopedics	58 (29%)	23.0–36.0
General surgery	63 (31.5%)	25.5–38.0
Neurosurgery	48 (24%)	18.0–30.0
Urology	31 (15.5%)	11.0–21.0
Surgery duration (min)	114.95 ± 68.95 (20–310)	—
Recovery duration (min)	49.45 ± 19.58 (20–120)	—
Pressure ulcer:		
No	163 (81.5%)	75.5–87.0
Yes	37 (18.5%)	13.0–24.5
Stage 1	25 (12.5%)	8.0–18.0
Stage 2	12 (6%)	3.0–10.0

**Table 2:** Frequency distribution of pressure injury location in patients.

Position	Injury location	Frequency	Percent
Supine	Sacrum	5	2.5
	Heel	3	1.5
	Leg	1	0.5
Prone	Patella	9	4.5
	Check	6	3
	Breast	2	1
	Eye	2	1
	Ear	1	0.5
	Toe	1	0.5
Lateral	Condyle	12	6
	Crest iliac	11	5.5
	Ear	6	3
	Malleolus	4	2
Lithotomy	Condyle	5	2.5
	Sacrum	3	1.5
	Scapula	2	1

To assess the construct validity of the measurement instrument, confirmatory factor analysis was conducted using AMOS software. The conceptual model included three latent factors: preoperative risk factors, intraoperative risk factors, and postoperative risk factors, and explained 69.933% of the total variance. The regression coefficients along with reliability and validity indices are shown in Table 3.

**Table 3:** Regression coefficients, along with reliability and validity indices.

Perioperative risk factor	Variable	Standardized regression weights	Unstandardized regression weights	S.E.	*t‐*value	*p*	AVE	CR	Cronbach's alpha
Preoperative risk factor	Mobility	0.652	1				0.452	0.83	0.84
	Nutritional state	0.607	0.626	0.08	7.66	< 0.01			
	BMI	0.555	1.187	0.17	7.02	< 0.01			
	Weight loss	0.683	0.767	0.09	8.43	< 0.01			
	Age	0.639	1.298	0.16	7.99	< 0.01			
	Comorbidity	0.857	3.266	0.33	10.05	< 0.01			
Intraoperative risk factor	ASA score	0.733	1				0.302	0.71	0.62
	Anesthesia	0.412	0.82	0.15	5.62	< 0.01			
	Temperature change	0.725	1.033	0.10	10.01	< 0.01			
	Hypotension change	0.785	1.09	0.10	10.87	< 0.01			
	Moisture	0.468	0.679	0.11	6.36	< 0.01			
	Surface/motion	0.175	0.27	0.12	2.35	0.019			
	Position	0.133	0.294	0.16	1.80	0.072			
Postoperative risk factor	Length of perioperative duration	0.783	1				0.690	0.82	0.83
	Blood loss	0.876	1.352	0.11	12.52	< 0.05			

*Note:* Standardized and unstandardized regression weights are presented for each item. *p* values were obtained from confirmatory factor analysis using AMOS. Statistical significance was set at *p* < 0.05.

Abbreviations: AVE = average variance extracted, CR = composite reliability, S.E. = standard error.

Based on the results in Table 3, it is observed that the factor loadings for all items, except for the items “position” and “surface/motion”, were higher than 0.4. To assess reliability, Cronbach's alpha and CR indices were used. The Cronbach's alpha values for preoperative, intraoperative, and postoperative risk factors were 0.84, 0.62, and 0.83, respectively. Additionally, the CR values for these three components were 0.83, 0.71, and 0.82, respectively, which are considered acceptable for these factors. The AVE index was used to evaluate convergent validity. The lowest AVE value was 0.30 for intraoperative risk factors, and the highest value was 0.69 for postoperative risk factors.

**Figure 1:**
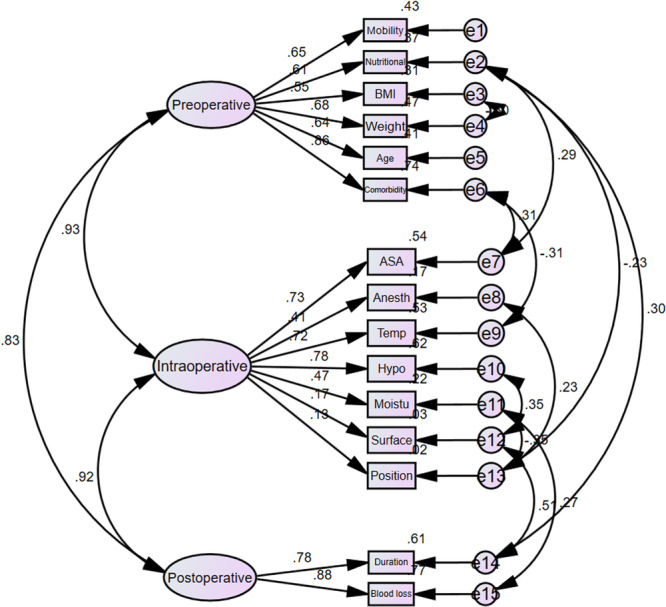
Standardized factor loadings for the confirmatory factor analysis (CFA) measurement model of the Munro pressure ulcer risk assessment scale (*n* = 200).

**Figure 2:**
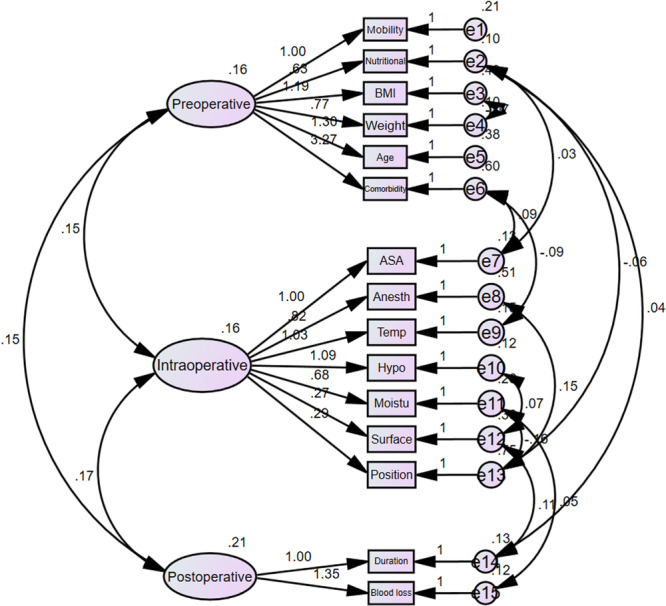
Unstandardized factor loadings for the confirmatory factor analysis (CFA) measurement model of the Munro pressure ulcer risk assessment scale (*n* = 200).

To evaluate the model fit, the Goodness of Fit Statistics were obtained as follows: the Root Mean Square Error of Approximation (RMSEA) was 0.112, the Root Mean Square Residual (RMR) was 0.039, the Comparative Fit Index (CFI) was 0.88, the Goodness of Fit Index (GFI) was 0.85, and the Adjusted Goodness of Fit Index (AGFI) was 0.77. Additionally, the *χ*² to degrees of freedom ratio value (*χ* ^2^/df) was 3.48. Figures 1, 2 demonstrate the standardized and unstandardized factor loadings for the components of the Munro scale in the confirmatory factor analysis measurement model.

The distribution of patients based on their risk of developing pressure ulcers, as assessed by the Munro scale, was examined across the three surgical phases: preoperative, intraoperative, and postoperative. In the preoperative phase, 15.5% of patients were classified as high risk, 55.5% as moderate risk, and 29% as low risk. Pressure ulcers subsequently developed in 64.52% of the high‐risk group, 12.61% of the moderate‐risk group, and 5.17% of the low‐risk group. During the intraoperative phase, 26% of patients fell into the high‐risk category, 68.5% into moderate risk, and 5.5% into low risk. Ulcers occurred in 46.15% of the high‐risk group, 9.49% of the moderate‐risk group, and none in the low‐risk group. In the postoperative phase, the same distribution was observed: 26% high risk, 68.5% moderate risk, and 5.5% low risk. Postoperative pressure ulcers developed in 48.08% of high‐risk patients, 8.76% of moderate‐risk patients, and none of the low‐risk patients (Table 4).

**Table 4:** Frequency distribution of patients based on the risk of pressure ulcer development according to the Munro Scale and the presence of pressure injury.

Stage	Risk category	Pressure ulcer		*p* value*
		No	Yes	
Preoperative Munro score total	Low risk	55 (94.83)	3 (5.17)	< 0.001
	Moderate risk	97 (87.39)	14 (12.61)	
	High risk	11 (35.48)	20 (64.52)	
Intraoperative Munro score total	Low risk	11 (100)	0 (0)	< 0.001
	Moderate risk	124 (90.51)	13 (9.49)	
	High risk	28 (53.85)	24 (46.15)	
Postoperative Munro score total	Low risk	11 (100)	0 (0)	< 0.001
	Moderate risk	125 (91.24)	12 (8.76)	
	High risk	27 (51.92)	25 (48.08)	

* *χ* ^2^ test.

To evaluate the diagnostic value of the Munro Scale in predicting the occurrence of pressure ulcers, two scenarios were considered. In the first scenario, only the high‐risk level on the Munro scale was regarded as indicative of pressure ulcer development. In the second scenario, both high and moderate‐risk levels were treated as predictive of pressure ulcer occurrence. The distribution of patients based on actual pressure ulcer presence and the predicted classifications according to the Munro scale is presented in Table 5.

**Table 5:** Distribution of patients based on the presence of pressure injury and predicted risk according to the Munro scale.

Scenario		True		Total
		Yes	No	
High risk = positive	Yes	25	27	52
	No	12	136	148
Moderate + high risk = positive	Yes	37	152	189
	No	0	11	11
Total		37	163	200

It can be observed that out of 37 patients who developed pressure ulcers, the Munro scale correctly identified 25 patients in Scenario 1 and all 37 patients in Scenario 2 as having pressure ulcers. Conversely, among the 163 patients without pressure ulcers, the Munro scale correctly classified 136 patients in Scenario 1 and 11 patients in Scenario 2. Based on these results, the diagnostic performance indices for both scenarios are presented in Table 6.

**Table 6:** Diagnostic accuracy indices of the Munro scale in identifying pressure injuries.

Index	Scenario1	Scenario2
Sensitivity	0.676	1
Specificity	0.834	0.067
Accuracy	0.805	0.24
PPV (positive predictive value)	0.481	0.196
NPV (negative predictive value)	0.919	1

## 4 Discussion

Pressure injuries remain one of the most common and costly postoperative complications, imposing a substantial burden on patients and healthcare systems [24]. In the present study, 37 of 200 patients (18.5%) developed pressure injuries, which were predominantly classified as Stage I and Stage II based on the National Pressure Injury Advisory Panel (NPIAP) classification system. The Munro pressure injury risk assessment scale for perioperative patients, encompassing nearly all risk factors identified in systematic reviews, offers a comprehensive perioperative risk evaluation tool [7, 25, 26]. In this study, the Persian adaptation of the Munro demonstrated strong psychometric properties overall, particularly in its preoperative and postoperative domains.

Validity denotes the extent to which an instrument accurately measures the construct it is intended to assess. In this study, we examined three forms of validity: face validity, content validity, and construct validity.

Face validity was evaluated by consulting surgical team members as subject‐matter experts to ensure that each item was clear and comprehensible. Content validity was assessed both qualitatively through expert feedback on relevance and comprehensiveness and quantitatively using Lawshe's method. The resulting content validity ratio was calculated using Lawshe's method. All items scored between 0.71 and 1.00 (threshold: > 0.62 for *N* = 10 experts).

Construct validity was appraised via CFA of the instrument's three‐section risk‐factor model (preoperative, intraoperative, postoperative). Model fit was evaluated using RMSEA, CFI, GFI, and *χ*²/df indices. While some indices, such as *χ²*/df and RMR, were within acceptable ranges, the RMSEA exceeded the recommended threshold, and CFI and GFI were below ideal values, indicating a moderate overall model fit. Convergent validity was assessed by calculating the AVE for each component. AVE values were 0.53 (preoperative), 0.30 (intraoperative), and 0.69 (postoperative), suggesting satisfactory convergence in the preoperative and postoperative domains but a relative weakness in the intraoperative section, likely due to lower factor loadings for “position” and “surface/motion.”

Similar to our findings, a study conducted to culturally adapt the Munro scale into Turkish also reported acceptable construct validity. However, in that study, the initial CFA demonstrated fit indices below the recommended thresholds, which led the researchers to perform exploratory factor analysis (EFA). Despite the suboptimal CFA results, the authors ultimately concluded that the scale achieved acceptable construct validity [14].

Both Cronbach's alpha and CR indices exceeded 0.80 for the preoperative (Cronbach's alpha = 0.84; CR = 0.83) and postoperative (Cronbach's alpha = 0.83; CR = 0.82) sections, indicating high internal consistency and reliability [27]. In line with our findings, a Brazilian cultural adaptation study of the Munro Scale also reported acceptable reliability, with Cronbach's alpha ranging from 0.82 to 0.86 and intraclass correlation coefficients between 0.75 and 0.91, supporting the overall stability of the scale across different populations [20].

Despite these strengths, the intraoperative subscale exhibited weaker measurement properties. Cronbach's alpha (0.62) and composite reliability (0.71) fall below thresholds commonly considered acceptable related to two items “position” and “surface/motion”. This finding mirrors Li et al.'s 2018 validation work, where removal of these items improved reliability metrics [16]. Nevertheless, proper patient positioning is a well‐established preventive measure against pressure ulcer development [28, 29, 30].

This apparent contradiction may be explained by an imbalance in the sample sizes across different positioning categories or by inconsistent adherence to safety protocols related to patient positioning in certain postures, such as the lithotomy and lateral positions. In our study, the lower factor loadings for these items and the resulting decrease in reliability for the intraoperative risk factor section may reflect these issues. These findings suggest that while these items are clinically important, their measurement properties within the scale may be affected by variability in practice and sample distribution. Additionally, in the present study, although the mean risk score for the “Position” item was higher in the supine position, the incidence of pressure injuries was greater in the lithotomy and lateral positions. This finding may indicate insufficient awareness or inconsistent adherence by nursing staff to proper patient positioning protocols in these two positions. In other words, despite the higher risk score in the supine position, localized pressure and duration in the lithotomy and lateral positions contributed to a higher occurrence of pressure injuries. This highlights the importance of staff education and standardization of patient positioning in the operating room and suggests that risk assessment should be interpreted in conjunction with adherence to positioning protocols.

In this study, the diagnostic performance of the Munro scale was further evaluated by calculating its sensitivity, specificity, and overall accuracy in two scenarios. Under the first scenario, where only patients classified as high risk were deemed positive for pressure injury, the tool achieved an overall accuracy of 80.5%, correctly classifying more than four‐fifths of cases. In the second scenario, accuracy fell to 24%, but sensitivity reached 1.00, indicating that every patient with a pressure ulcer was successfully identified. Actually, the second scenario is therefore preferable in settings where failing to detect even a single pressure ulcer is unacceptable, despite the attendant increase in false positives. Conversely, the first scenario is more appropriate when diagnostic precision is paramount, and resources (such as staff or equipment) limit the capacity to implement preventive measures broadly; concentrating on high‐risk patients in this context ensures a more efficient allocation of scarce resources.

Strengths of this study include rigorous forward backward translation, cultural adaptation for an Iranian clinical context, and comprehensive psychometric evaluation encompassing reliability, construct validity, convergent validity indices, and diagnostic tests. Also, six operating room nurses received standardized training for marking scales for increasing inter‐rater reliability. However, the study has several limitations. No established comparator instrument, such as the Braden scale, was used alongside the Munro scale, limiting the ability to directly evaluate predictive performance. In addition, the distribution of patients across different surgical positions, particularly lithotomy and lateral, was uneven, which may have influenced factor loadings and the observed incidence of pressure injuries. Finally, variability in adherence to proper patient positioning protocols among nursing staff may have contributed to fluctuations in the results, highlighting the potential impact of clinical practice on the measurement properties of the scale. Future studies are recommended to include multiple centers, standardized comparator instruments, and targeted staff training in patient positioning and marking surface/motion scales to further validate the Munro scale and optimize its predictive utility in perioperative settings.

## 5 Conclusion

In conclusion, the Persian version of the Munro scale demonstrates strong psychometric credentials overall, particularly in its preoperative and postoperative domains. The intraoperative subscale demands further refinement through item redefinition, rater calibration, and potentially enriched response anchors to capture the nuances of surgical positioning and support surfaces. Future research should pursue multicenter trials across diverse surgical specialties, compare against emerging risk tools, and evaluate the impact of targeted training on both item performance and patient outcomes. Such efforts will enhance the scale's utility as a cornerstone of pressure ulcer prevention in perioperative nursing practice.

## Author Contributions

**Leila Sadati:** conceptualization, investigation, methodology, writing – review and editing, and writing – original draft. **Sahar Karami:** investigation, conceptualization, and validation. **Nasim Jamshid Malekara:** conceptualization, writing – original draft, project administration, supervision, writing – review and editing. **Rana Abjar:** visualization, software, and formal analysis. **Cassendra A. Munro:** writing – review and editing, supervision, and methodology. **Niloofar Hajati:** resources and investigation.

## Funding

The authors received no specific funding for this work.

## Ethics Statement

This study was approved by the Ethical Review Committee of Alborz University of Medical Sciences with IR.ABZUMS.REC.1403.114 ethics code.

## Consent

All participants provided written informed consent prior to their inclusion in the study.

## Conflicts of Interest

The authors declare no conflicts of interest.

## Transparency Statement

The lead author, Nasim Jamshid Malekara, affirms that this manuscript is an honest, accurate, and transparent account of the study being reported; that no important aspects of the study have been omitted; and that any discrepancies from the study as planned (and, if relevant, registered) have been explained.

## Data Availability

The data that support the findings of this study are available on request from the corresponding author. The data are not publicly available due to privacy or ethical restrictions.
